# Meiotic Regulation of TPX2 Protein Levels Governs Cell Cycle Progression in Mouse Oocytes

**DOI:** 10.1371/journal.pone.0003338

**Published:** 2008-10-03

**Authors:** Stéphane Brunet, Julien Dumont, Karen W. Lee, Kazuhisa Kinoshita, Pascale Hikal, Oliver J. Gruss, Bernard Maro, Marie-Hélène Verlhac

**Affiliations:** 1 UMR7622, Université Pierre et Marie Curie/CNRS, Bat. C, 5e, 9 quai Saint Bernard, Paris, France; 2 Chromosome Dynamics Laboratory, RIKEN, Wako, Japan; 3 Zentrum für Molekulare Biologie der Universität Heidelberg (ZMBH), Heidelberg, Germany; 4 Department of Cell and Developmental Biology, Tel Aviv University, Ramat University, Tel Aviv, Israël; University of Edinburgh, United Kingdom

## Abstract

Formation of female gametes requires acentriolar spindle assembly during meiosis. Mitotic spindles organize from centrosomes and via local activation of the RanGTPase on chromosomes. Vertebrate oocytes present a RanGTP gradient centred on chromatin at all stages of meiotic maturation. However, this gradient is dispensable for assembly of the first meiotic spindle. To understand this meiosis I peculiarity, we studied TPX2, a Ran target, in mouse oocytes. Strikingly, TPX2 activity is controlled at the protein level through its accumulation from meiosis I to II. By RNAi depletion and live imaging, we show that TPX2 is required for spindle assembly via two distinct functions. It controls microtubule assembly and spindle pole integrity via the phosphorylation of TACC3, a regulator of MTOCs activity. We show that meiotic spindle formation *in vivo* depends on the regulation of at least a target of Ran, TPX2, rather than on the regulation of the RanGTP gradient itself.

## Introduction

The assembly of a functional spindle is critical for accurate chromosome segregation. At the end of meiosis, oocytes undergo two successive divisions (meiosis I and II) to form haploid gametes. During these divisions, meiotic spindles must ensure successively the segregation of homologous chromosomes and sister chromatids. Homologous chromosome missegregation in meiosis I is a major source of embryonic aneuploidy in mammals, and accounts for most spontaneous abortion and birth defects in human [1], [2]. This peculiarity of meiosis I is poorly understood at the molecular level and the mouse oocyte constitutes the model of choice for its study. However, not much work has been performed on this model to understand the discrepancy between meiosis I and II spindle assembly. Therefore, understanding the mechanisms that govern the formation of meiotic spindles in mammals remains an important and challenging goal for developmental and cell biologists.

Mouse oocytes lack centrioles, instead, they contain multiple microtubule-organizing centres (MTOCs, [3]). After nuclear envelope breakdown in meiosis I, microtubules emanate from these MTOCs as an unorganised mass around the chromosomes. MTOCs and microtubules are then progressively sorted out into a bipolar barrel-shaped spindle [4]. Strikingly, this process is restricted to the close vicinity of chromosomes and takes place in the absence of stable kinetochore-microtubule interactions [5]. In mitosis, chromosomes contribute to spindle formation independently of their kinetochores by locally activating spindle assembly factors (for review see [6]). This effect relies on the small GTPase Ran. The active GTP-bound form of Ran (Ran-GTP) is produced on the chromatin and a Ran-GTP gradient centred on chromosomes is generated (for review see [7]). Within the gradient, Ran-GTP activates spindle assembly factors by releasing them from the nuclear transport receptors, importins α and β.

In mouse oocytes, the Ran-GTP gradient is present during both meiotic divisions. Alteration of Ran-GTP levels impairs meiosis II spindle assembly but does not prevent the formation of a functional spindle during meiosis I. It only delays the setting up of its bipolarity and affects its size [8]. This discrepancy raises the possibility that Ran-GTP dependent mechanisms are regulated differently during these two divisions. On the other hand, when chromosomes are removed from mouse oocytes, MTOCs and microtubules can self-organize into bipolar structures [9]. Hence, such a self-organization mechanism, independent of Ran-GTP, may act to a different extent during meiosis I and II.

To understand the mechanisms at play, we have investigated the role and regulation of a central Ran target TPX2 (Targeting Protein for the *Xenopus* kinesin xklp2; [10]) during meiotic divisions in mouse oocytes. In somatic cells, TPX2 is cell-cycle regulated, it accumulates in the nucleus during interphase, on spindle poles during mitosis, and is degraded at mitotic exit [11], [12], [13]. It is essential for spindle assembly, but the molecular mechanisms at play are complex and not fully unravelled. TPX2 induces a Ran-GTP dependent microtubule nucleation in the vicinity of chromosomes [14]. It also participates in spindle pole organization [12], [15]. TPX2 has been shown to bind and activate the kinase Aurora A (for review see [16]), an essential regulator of centrosome and spindle pole assembly (for review see [17]). On the other hand, Aurora A phosphorylates TACC3 (Transforming Acidic Coiled-Coil protein). This phosphorylation may contribute to the stabilization of centrosomal microtubules, presumably by locally activating the microtubule stabilizing protein TOG (for review see [18]).

Here we show that TPX2 is necessary for acentriolar spindle assembly in mouse oocytes. TPX2 activity is tightly regulated at the protein level during the two successive meiotic divisions. In prophase I, the complete absence of TPX2 depends on the activity of the anaphase-promoting complex/cyclosome (APC/C) activator, cdh1. TPX2 accumulates from the onset of meiosis I to the metaphase arrest of meiosis II. These increasing levels of TPX2 coincide with those required for spindle assembly in meiosis I and II. In addition, our data show that TPX2 controls acentriolar spindle poles integrity via phosphorylation of TACC3 at the MTOCs.

## Materials and Methods

### Mouse oocyte collection, culture and microinjection

Oocytes were collected from 11-week-old OF1 female mice, cultured and microinjected as described [19]. Oocytes were maintained at the GV stage in M2+BSA medium supplemented with 1 µM of milrinone [20]. *In vitro* synthesized cRNA were microinjected into the cytoplasm of GV oocytes. Injected oocytes were kept in M2+BSA medium supplemented with milrinone for various time periods (2 or 5 h). For long-term incubation, oocytes were cultured in MEM with 20% foetal calf serum in a 5% CO2 humidified incubator at 37°C as described [20]. The resumption of meiotic maturation (GVBD) was triggered upon release of the oocytes into a milrinone-free M2+BSA medium.

### Plasmid construction, *in vitro* transcription of synthetic RNA and siRNA sequences

The YFP-hTPX2 was sub-cloned into pRN3 at the Not I site. The YFP-TPX2ΔN was produced by deletion of the full-length hTPX2 from its N-terminal 1 to 118 amino acids. The pRN3-CyclinB1-GFP and pRN3-Histone-RFP constructions have been described previously [21], [22]. The *in vitro* synthesis of capped RNA was performed as described [19].

To knock-down Cdh1, we used the antisense morpholino-oligonucleotides (Gene Tools, LLC) as described [20], [23]. To knock-down TPX2 expression, we used the corresponding double-stranded siRNA at 20 µM (Invitrogen): 5′-GGUUCCAGUAGAAGCUGCAUCUAUA for siTPX2 and 5′-GGUUGACAAGAGUCGCUACUCUAUA for siControl (siCo). To knock-down TACC3 expression, we used the corresponding double-stranded siRNA at 20 µM (Invitrogen): 5′-GAGAAACAGAGGGAACUAAAGGAAA for siTACC3 and a medium GC duplex siRNA for control (12935-300; Invitrogen).

The transgenic ZP3-β5Tubulin-GFP mouse line was produced at the Transgenic Institute/SEAT-UPS 44 (Villejuif, France) and will be further described in Lee *et al.* (*in preparation*).

### Videomicroscopy

Time-lapse images were acquired using a Photometrics CCD camera (CoolSnap HQ2) mounted on a Leica DMI600B microscope using a Leica HC PL APO 20X/0.7 NA objective enclosed in a thermostatic chamber (Life Imaging Services). The Metamorph software 7.0 (Universal Imaging) and ImageJ (NIH) were used for image analysis. To measure YFP-TPX2 expression, movies simultaneously imaging oocytes expressing both high and low levels of YFP-TPX2 were used and the mean YFP intensity over the background for each oocyte was estimated.

### Immunofluorescence

Immunofluorescence of mouse oocytes was performed as described [5]. For microtubules, we used a rat monoclonal antibody against tyrosinated α-tubulin (YL1/2). TACC3 is phosphorylated on Ser626 in *Xenopus* by Aurora A, this serine is conserved in human, *Drosphophila* and mouse. It corresponds to Ser347 in mouse TACC3. For anti-phospho-Ser626 xTACC3, we used a rabbit polyclonal antibody described in [24]. Images were acquired with a Leica SP5 confocal microscope using a Plan APO 40X/1.25 NA objective. Z-series were performed with a Z-step of 1 µm and maximum projections of Z planes including the spindle region are displayed. To compare the intensity of P-TACC3 labelling, oocytes from different experimental conditions were fixed at the same time and analyzed under the confocal microscope using the same settings. For each oocyte, a maximal projection of 10 planes was performed. This projection was enlarged by 200% and the intensity of the P-TACC3 labelling in a 1.26 µm^2^ square region was measured from different experimental conditions.

The measure of spindle length in different conditions was performed using the Volocity 4.1 software (Improvision) to have a 3D reconstruction of spindles after fixation (for this we used the Z-stack obtained on the Leica SP5 confocal microscope).

### Immunoblotting

Immunoblotting of mouse oocytes was performed as described [25]. The anti-hTPX2 antibody has been described [14]. The anti-xTACC3 and anti-pSer626-xTACC3 antibodies have been described [24]. The anti-cdh1 has been described (Reis et al., 2007).

## Results

### Cdh1–dependent accumulation of TPX2 in maturing mouse oocyte

**Figure 1:**
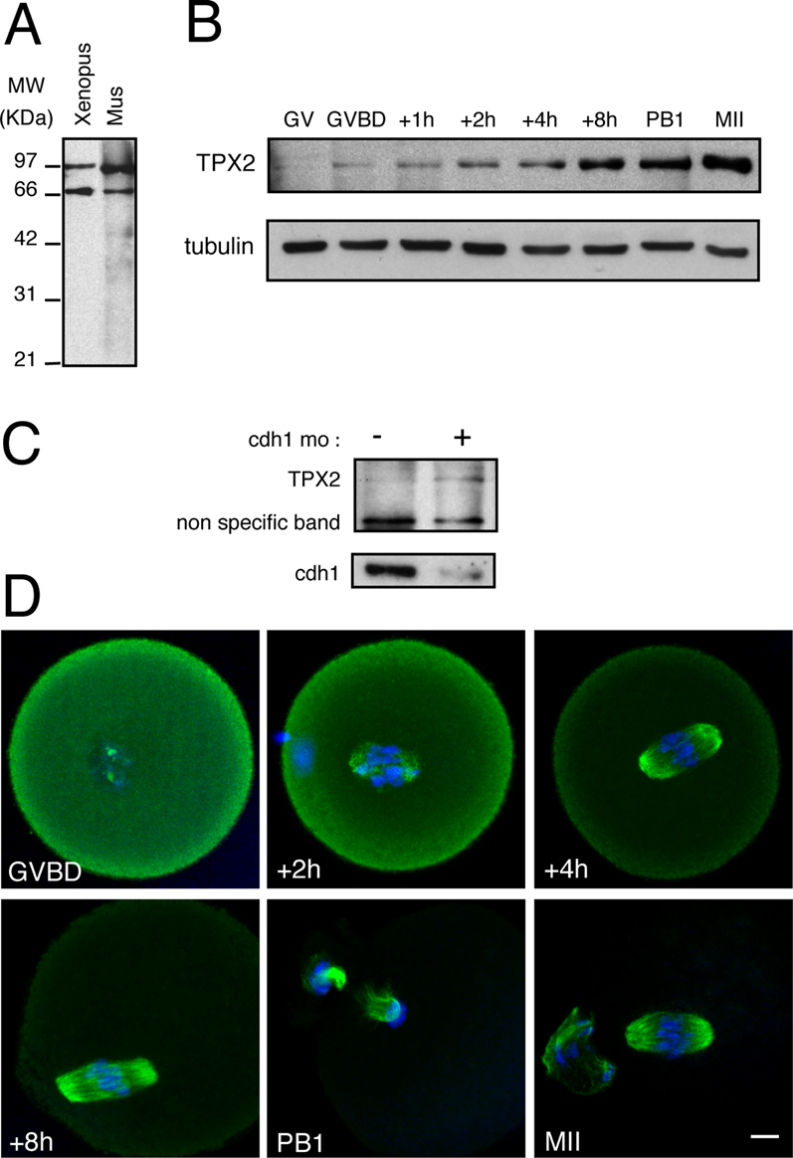
Characterization of TPX2 during mouse oocyte meiotic maturation. A: Mouse oocytes (n = 200) and *Xenopus* metaphase II oocyte lysate (equivalent to 1/4 of oocyte) were immunoblotted using an anti-TPX2 antibody. The upper band corresponds to TPX2. The lower band is non-specific [11]. B: TPX2 accumulates progressively during meiotic maturation. For each time point, 140 mouse oocytes were immunoblotted using anti-TPX2 (only the TPX2 specific band is presented) and anti-α-tubulin antibodies. C: Cdh1 controls TPX2 levels at GV (Prophase I). Western blot analysis of TPX2 and cdh1 in control oocytes and cdh1 morpholino injected oocytes (n = 100 for each group). D: Localization of TPX2 during meiotic maturation. Oocytes were double stained for TPX2 (green) and nucleic acids (blue) at the times indicated relative to GVBD. In meiosis I and II, TPX2 decorates the spindle microtubules. Scale bar is 10 µm.

TPX2 expression was first analyzed by immunoblot during meiotic maturation from prophase I to the metaphase II arrest using an antibody directed against *human* TPX2 (Fig 1A). TPX2 accumulated after germinal vesicle breakdown (GVBD, equivalent to nuclear envelope breakdown) until the metaphase arrest of meiosis II (Fig 1B). However, in contrast to somatic cells, TPX2 was absent during prophase. TPX2 localization was then examined by immuno-fluorescence staining (Fig 1D). We did not observe any specific staining of TPX2 in GV oocytes (data not shown). Around GVBD (Fig 1D panel GVBD), TPX2 was observed as punctuate structures around the chromosomes reminiscent of MTOCs. Once the bipolar spindle was formed, TPX2 decorated spindle microtubules and was enriched at the spindle poles. Consistent with the accumulation of TPX2 during meiotic maturation, we observed a progressive increase in the signal to noise ratio of the TPX2 labelling (Fig 1D).

In mouse oocytes, the APC^cdh1^ triggers cyclin B1 proteolysis during prophase and prometaphase I, contributing to the prophase arrest and slowing down the progression of meiosis I [20], [23]. We reasoned that an APC^cdh1^ -dependent degradation could similarly govern TPX2 protein levels in maturing oocytes. Therefore, we examined the ability of oocytes to accumulate TPX2 in prophase I following microinjection with the antisense morpholino directed against cdh1 [20], [23]. Oocytes were maintained for 24 hours in prophase after microinjection and TPX2 expression was analyzed by immunoblot. In these conditions, endogenous TPX2 was detected in cdh1-knockdown prophase I-arrested oocytes but not in controls (Fig 1C), showing that TPX2 accumulation indeed is antagonized by APC^cdh1^ activity in oocytes.

### TPX2 premature over-expression is deleterious for Meiosis I completion

In order to assess the functional significance of this accumulation of TPX2, we expressed a YFP-tagged TPX2 (YFP-TPX2) at different levels in the oocyte. Prophase-arrested oocytes were microinjected with a cRNA encoding a YFP-tagged human TPX2. Meiotic maturation was then allowed to proceed and spindle assembly was analyzed by time-lapse videomicroscopy, taking advantage of the association of YFP-TPX2 with spindle microtubules.

**Figure 2:**
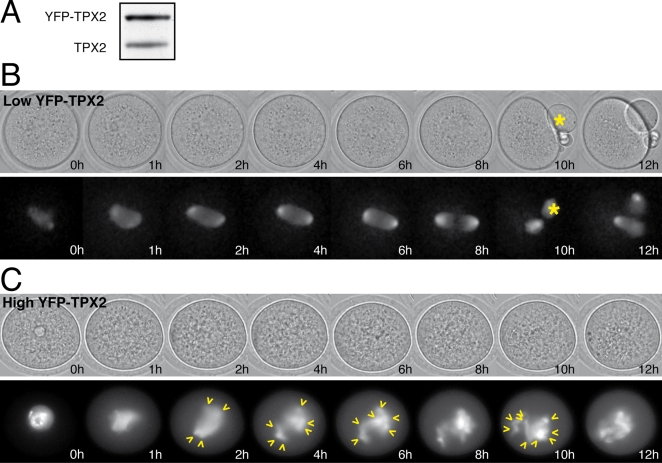
TPX2 overexpresssion induces an arrest in meiosis I. A: Immunoblot analysis of endogenous (TPX2) and exogenous (YFP-TPX2) protein levels in metaphase II oocytes (n = 72) expressing low levels of YFP-TPX2. B and C: Representative time-lapse videomicroscopy of oocytes expressing low (B; n = 78) or high (C; n = 29) levels of YFP-TPX2. Upper panels are the bright field images of the oocyte and the lower panels show the fluorescent YFP signal at the corresponding time points (germinal vesicle breakdown is 0 h). While the polar body extrusion (asterisks) was normal in (B), it was blocked in (C). In this case, multipolar microtubules structures were observed (see arrowheads).

The expression of YFP-TPX2 at physiological levels, as determined by immunoblot analysis (Fig 2A), did not perturb meiotic maturation. In addition, YFP-TPX2 behaved like the endogenous protein, accumulating at spindle poles of meiosis I and meiosis II spindles (Fig 2B, video S1).

When over-expression reached around 6-fold above the physiological levels (by quantitative analysis of the YFP signal, see Experimental Procedures), only 24% of oocytes extruded a polar body 8 to 9 hours after GVBD, while 92% of the controls did. YFP-TPX2 accumulated in the germinal vesicle of oocytes in prophase I (Fig 2C). During meiosis I, spindle assembly was impaired: microtubules failed to sort and organize into a bipolar spindle and led to the formation of multipolar structures (Fig 2C, arrowheads and video S2). These observations suggest that premature expression of high levels of TPX2 interferes with proper spindle assembly and that progressive accumulation of TPX2 is essential to integrate its functions during meiotic maturation.

### TPX2 depletion impairs meiotic progression in a dose-dependent manner

**Figure 3:**
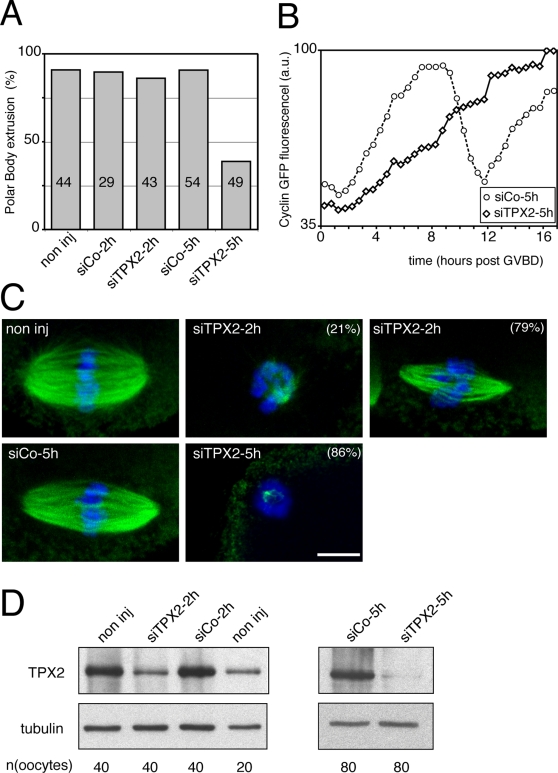
Meiotic maturation and spindle formation upon TPX2 depletion. A: Percentages of polar body extrusion after siRNA treatment. Non-injected (non inj), control and TPX2 siRNA-injected oocytes maintained in prophase for 2 or 5 hours (siCo-2h, siCo-5h, siTPX2-2h and siTPX2-5h, respectively) were analyzed. The total number of oocytes for each condition is indicated in the bar. B: Semi-quantitative analysis of the Cyclin B1-GFP fluorescence during meiotic maturation (a.u.: arbitrary units). For each time point, the fluorescence was normalized against the maximum value reached for each oocyte during meiotic maturation. The curves represent the mean curves extracted from 10 oocytes. C: Representative spindle morphology upon TPX2 depletion. Non-injected, siCo-5h and siTPX2-2h injected oocytes at metaphase II. SiTPX2-5h injected oocytes arrested in meiosis I. Both non-injected and siCo-5h injected oocytes showed similar barrel-shaped bipolar spindles. However, siTPX-2h injected showed either small aster around the chromosomes (21%) or a mini spindles (79%). siTPX2-5h injected oocytes, on the other hand, displayed more severe perturbation in spindle assembly with only few microtubules present around the chromosomes in most of the cases (86%). Microtubules are in green and chromosomes are in blue. Scale bar is 10 µm. D: Immunoblot analysis of TPX2 upon siRNA depletion. Both siCo-2h and siCo-5h showed similar TPX2 levels as the non-injected oocytes. SiTPX2-2h depleted about half of the TPX2 protein while the siTPX2-5h almost completely depleted the protein. The number of oocytes in each sample is indicated below the immunoblots. α-tubulin was used as loading control.

The effect of TPX2 depletion was then investigated. Oocytes injected with siRNA duplex oligonucleotides were maintained in prophase I for either two or five hours (referred to as siTPX2-2h and -5h, respectively), then meiotic maturation was allowed to proceed. While most of non-injected, control siRNA (siCo-2h and siCo-5h) and siTPX2-2h injected oocytes extruded the first polar body, more than 60% of the siTPX2-5h injected oocytes did not (Fig 3A and video S3 and S4). To test whether these oocytes were arrested in meiosis I, either siCo-5h or siTPX2-5h injected oocytes were co-injected in prophase with a cyclinB1-GFP encoding cRNA. This allowed the monitoring of cyclin B1 levels during meiotic maturation. In siCo-5h injected oocytes (Fig 3B), cyclin B1 accumulated during meiosis I, dropped around 9 hours after GVBD, when the first polar body was extruded and re-accumulated rapidly during meiosis II, as expected [21]. On the contrary, siTPX2-5h injected oocytes exhibited a constant accumulation of cyclin B1 (Fig 3B), indicating that meiosis I completion was inhibited.

### TPX2 depletion damages meiotic spindle morphology in a dose-dependent manner

Next, spindle structures were analyzed by immunofluorescence staining. The spindle morphology in siTPX2-5h injected oocytes arrested at meiosis I was abnormal. 86% of the oocytes contained only a few microtubules associated with the chromosomes and the remaining 14% contained only tiny bipolar spindles (Fig 3C and data not shown). Aberrant microtubule structures were also observed in siTPX2-2h injected oocytes arrested at meiosis II, in contrast to the meiosis II spindles of control oocytes. Small asters associated with the chromosomes or tiny bipolar spindles were observed in 21% and 79% respectively, of the siTPX2-2h injected oocytes.

We analyzed the efficiency of TPX2 depletion by immunoblot. TPX2 was substantially depleted in siTPX2-5h injected oocytes, while only about half of the protein was depleted in the siTPX2-2h injected oocytes (Fig 3D).

In addition, the specificity of the siRNA treatment was confirmed by coinjecting siTPX2 duplex oligonucleotides with a cRNA encoding a YFP-tagged human TPX2, which is not targeted by the siRNA. Under these conditions, the first polar body extrusion was restored up to 72% and both meiosis I and II spindle morphology appeared normal.

### Dynamics of meiotic spindle assembly upon TPX2 depletion

**Figure 4:**
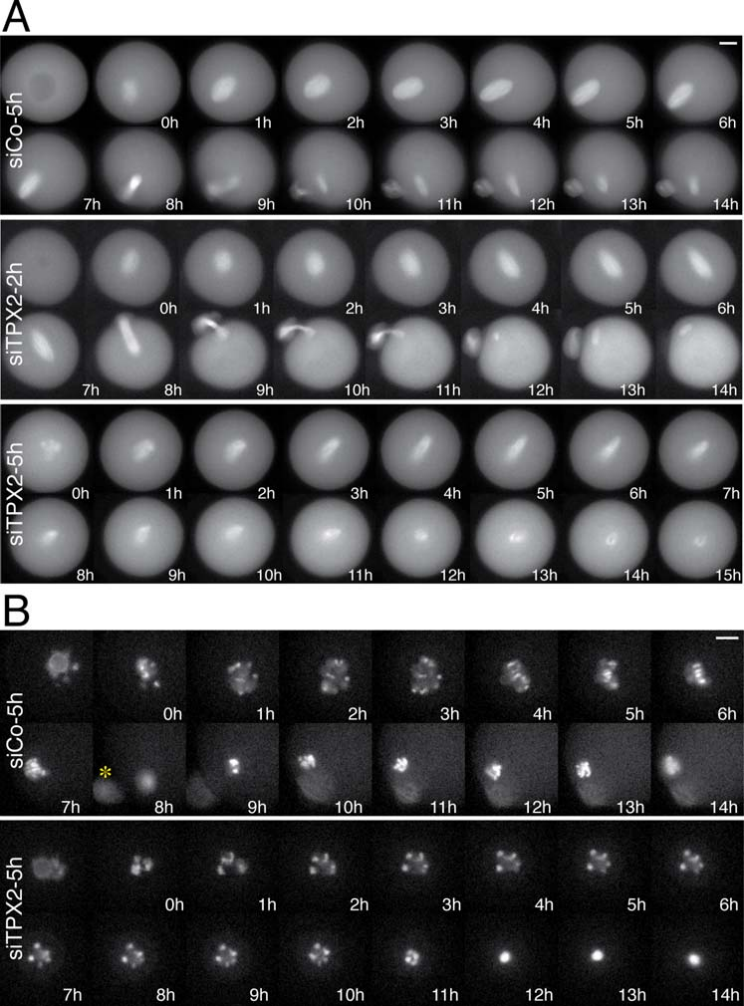
Microtubules and chromosomes behaviour upon TPX2 depletion. A: Representative sequence of fluorescent images showing the microtubule organization in siCo-5h (top panel), siTPX2-2h (middle panel) and siTPX2-5h (bottom panel) injected oocytes expressing a GFP-tagged β-tubulin. Both siCo-5h and siTPX2-2h injected oocytes extruded the polar bodies. However, siTPX2-2h injected oocyte formed small spindle after the polar body extrusion (see panel 12 h). The siTPX2-5h depletion blocked polar body extrusion and the bipolar spindle formed started to collapse three to five hours after GVBD (panels 5 h–15 h). Times are relative to GVBD. B: Representative sequence of fluorescent images showing the morphology and movement of the chromosomes in siCo-5h (upper panel) or siTPX2-5h (bottom panel) injected oocytes expressing RFP-histone H2B. While the chromosomes progressively aligned onto the metaphase plate in the siCo-5h injected oocyte (panel 6 h), the chromosomes in the siTPX2-5h injected oocyte appeared to be stationary during the few hours after GVBD (panels 2 h–8 h). Times are relative to GVBD.

The dynamic process of spindle organization upon TPX2 depletion was analyzed by time-lapse videomicroscopy in maturing oocytes from a transgenic line expressing a GFP-tagged β-tubulin (Lee *et al.*, in preparation). The kinetics of meiosis I spindle assembly were similar in siTPX2-2h and control oocytes. However, after polar body extrusion, only tiny spindles formed in the siTPX2-2h oocytes (Fig 4A, middle panel and videos S3 and S4).

In siTPX2-5h injected oocytes, microtubules first organised after GVBD into a bipolar array as in control oocytes. Nevertheless, 3 to 5 hours after GVBD, this bipolar spindle started to collapse. The spindle shrank progressively and ended up as small asters (Fig 4A, lower panel).

The chromosome behaviour was also followed in these oocytes upon co-injection of a cRNA encoding an RFP-tagged histone in prophase. In control oocytes, individual chromosomes could be observed, especially during meiosis I, where they progressively aligned on the metaphase plate (video S5). In siTPX2-5h injected oocytes however, the chromosomes appeared to be immobile during the first few hours after GVBD (video S6). In addition, concomitant to the spindle collapse, they progressively aggregated and resulted in a single clump in the cytoplasm (Fig 4B, lower panel). Taken together, these data indicate that TPX2 is essential for meiotic spindle assembly, but at distinct thresholds of activity during meiosis I and meiosis II.

### TPX2 is essential for acentriolar spindle size and pole integrity

**Figure 5:**
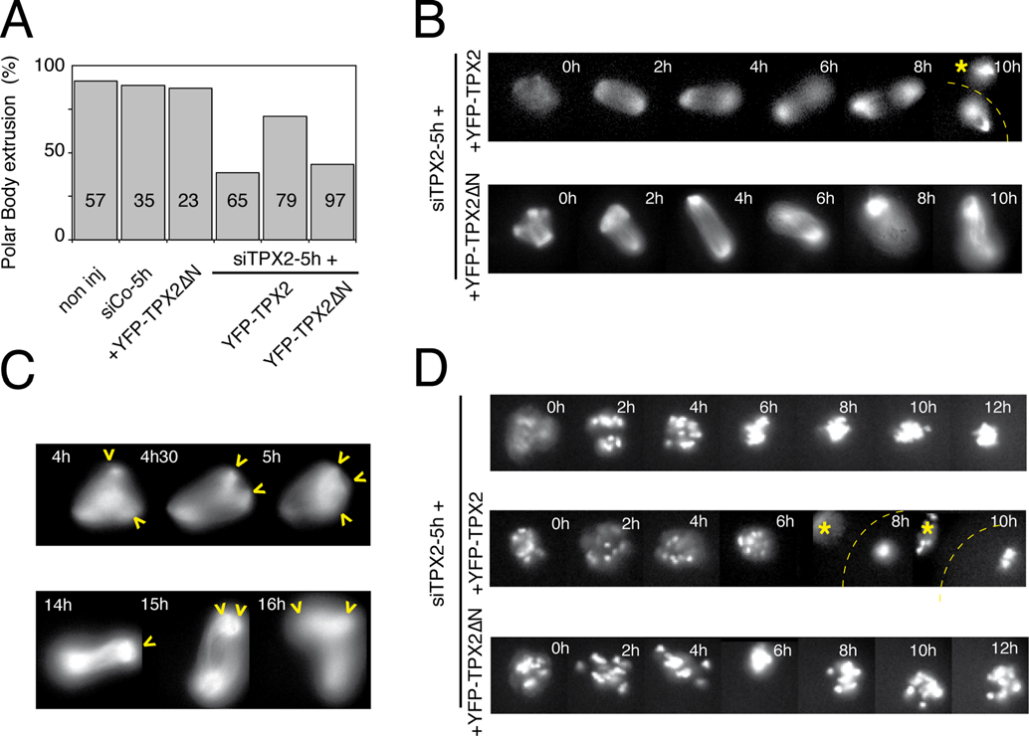
TPX2 activity relies on two distinct protein domains in the oocyte. A: Percentage of polar body extrusion in different experimental conditions. Non-injected, siTPX2-5h, YFP-TPX2ΔN injected oocytes as well as oocytes co-injected with siTPX2-5h and YFP-TPX2 or YFPTPX2ΔN were analyzed. The total number of oocytes examined is indicated in each bar. B: Representative sequence of fluorescent images showing the microtubules organization in siTPX2-5h injected oocytes, which were co-injected either with YFP-TPX2 (upper panel) or YFP-TPX2ΔN (lower panel). Both YFP-TPX2 and YFP-TPX2ΔN rescued the spindle collapse. Asterisk indicates the polar body and the dotted line shows the outline of the oocyte. C: Representative fluorescent images of siTPX2-5h-injected oocytes co-injected with YFP-TPX2ΔN show spindle poles splitting (arrowheads). D: Representative sequence of fluorescent images showing the chromosomes siTPX2-5h injected oocytes expressing RFP-histone H2B alone (top panel), co-injected with either YFP-TPX2 (middle panel) or YFP-TPX2ΔN (bottom panel). The asterisk indicates the polar body and the dotted line shows the outline of the oocyte. Times are relative to the GVBD.

In mitosis, TPX2 is essential to promote microtubule assembly [11]. Both C- and N-terminal domains of TPX2 have been proposed independently to support this function [26], [27]. To explore the mechanisms at play in the oocytes, we generated a YFP fusion of a truncated form of hTPX2 lacking the 118 first amino acids. The ability of this protein (YFP-TPX2ΔN) to rescue TPX2 depletion in the oocyte was investigated. The expression of YFP-TPX2ΔN alone did not alter meiotic maturation (Fig 5A). Upon TPX2 substantial depletion, YFP-TPX2ΔN was not able to rescue polar body extrusion in contrast to the full-length YFP-TPX2 (videos S7 and S8). Only 40% of the siTPX2-5h oocytes co-injected with YFP-TPXΔN cRNA extruded the first polar body, while 70% of the TPX2 depleted oocytes expressing YFP-TPX2 did.

Taking advantage of the association of both YFP-TPX2ΔN and YFP-TPX2 [27] with microtubules, the spindle dynamics were analyzed using time-lapse video microscopy. In contrast to the spindle shrinkage to a size of 6.3±2 µm observed upon TPX2 substantial depletion, large microtubule structures were observed upon co-injection with either YFP-TPX2 or YFP-TPX2ΔN (Fig 5B). Similarly, instead of a single chromosome clump observed upon TPX2 depletion (Figs 4B and 5D), individual chromosomes could be visualized upon co-injection with either YFP-TPX2 or YFP-TPX2ΔN (Figs 5B and D). The observations indicated that a large C-terminal part of the TPX2 was sufficient to promote microtubule assembly in the oocyte.

MII spindles in controls and in TPX2 depleted oocytes expressing YFP-TPX2 have similar lengths: 23±2 µm and 21±5 µm, respectively. In contrast, the spindle length in TPX2 depleted oocytes expressing YFP-TPX2ΔN reached 36±5 µm, which corresponds to a classical meiosis I spindle size [8]. In these oocytes however, spindle pole integrity appeared significantly reduced, resulting in some cases to the formation of transient tri- or multi-polar structures (Figs 5B and C and video S8). These observations suggested that the N-terminal domain of TPX2 was required to promote spindle pole integrity and robustness. In conclusion, both N- and C-terminal domains are at play through distinct mechanisms to fulfil TPX2 functions in the oocyte.

### TPX2 controls spindle pole organization via TACC3 phoshorylation

**Figure 6:**
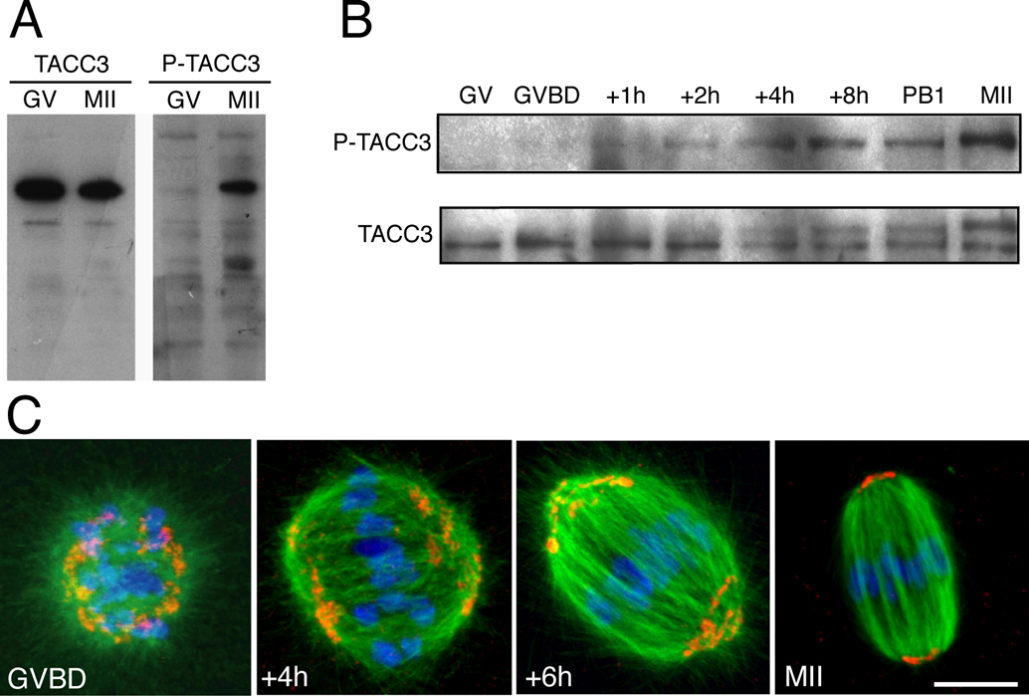
Characterization of TACC3 during mouse oocyte meiotic maturation. A: Immunoblot analysis of TACC3 and phosphorylated TACC3 (P-TACC3) in GV and metaphase II (MII) oocytes. Oocytes (n = 150) were immunoblotted using an anti-TACC3 antibody (left) and an antibody raised against xTACC3 phosphorylated on Ser626 (right). B: Immunoblot analysis of TACC3 and P-TACC3 during meiotic maturation. Oocytes (n = 140) were immunoblotted at the indicated time points. C: Localization of P-TACC3 during meiotic maturation. Oocytes were triple stained for P-TACC3 (red), microtubules (green) and nucleic acids (blue). The P-TACC3 staining becomes restricted to the spindle poles during meiotic maturation. Scale bar is 10 µm.

The N-terminus of TPX2 was previously demonstrated to bind and activate Aurora A during M-phase [26], [28], [29], [30]. Recently, Aurora A was shown to phosphorylate and activate the centrosomal protein TACC3, stimulating in turn microtubule assembly at the centrosomes [24], [31]. We reasoned that during oocyte meiotic maturation, in the absence of centrosomes, TPX2 could indirectly induce the phosphorylation and activation of TACC3 at MTOCs. TACC3 and its phosphorylated form on the conserved Ser347, which corresponds to Ser626 in Xenopus, were first analyzed by immunoblot. While TACC3 was present at constant levels during meiotic maturation (Fig 6A), the phosphorylated form of TACC3 (P-TACC3) accumulated from GVBD to the metaphase arrest of meiosis II (Fig 6B), concomitantly with the progressive accumulation of TPX2 (see Fig 1B). The localization of P-TACC3 in the oocyte was examined. During GVBD, P-TACC3 decorated punctate structures at the center of microtubule asters emanating from the chromosomes. Once a bipolar spindle was formed, the punctate P-TACC3 labelling was restricted to spindle poles (Fig 6C), strongly suggesting that P-TACC3 associated with MTOCs in the oocyte.

**Figure 7:**
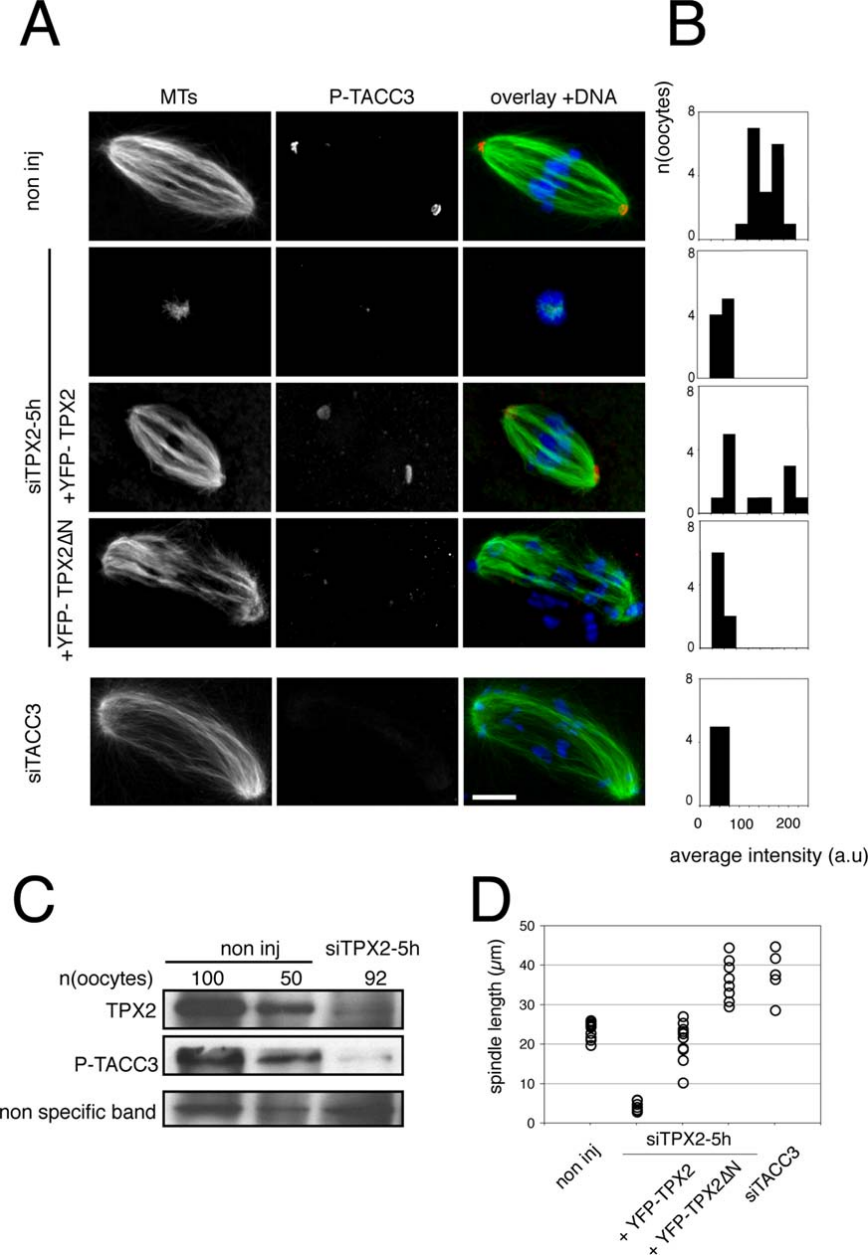
TACC3 is an indirect target of TPX2 in the oocyte. A: Localization of P-TACC3 upon TPX2 depletion. Oocytes were triple labelled for P-TACC3 (red), microtubules (green) and nucleic acids (blue). P-TACC3 staining is absent in siTPX2-5h, siTPX2-5h co-injected with YFP-TPX2ΔN and siTACC3 oocytes. Scale bar is 10 µm. B: Semi-quantitative analysis of the distribution of the average P-TACC3 fluorescence intensity in individual oocytes. C: Western blot analysis of P-TACC3 and TPX2 levels upon TPX2 depletion. Both TPX2 and P-TACC3 proteins are significantly reduced after siTPX2-5h treatment. Numbers of oocytes used were indicated in each lane. The TPX2 non-specific band (lower band in Fig 1A) is used as a loading control. D: Spindle length measurements in the different experimental conditions. TPX2 depletion induces a dramatic reduction of the spindle size. The size is restored upon co-injection with YFP-TPX2. Large spindles, characteristic of meiosis I, did form in TPX2 depleted oocytes co-injected with YFP-TPX2ΔN as well as in TACC3 depleted oocytes. Each circle represents an individual measurement.

The effect of TPX2 silencing on TACC3 phosphorylation was then investigated. In non-injected control oocytes, the spindle poles showed pronounced staining of P-TACC3, while this puntacte P-TACC3 labelling was hardly detected in TPX2-depleted oocytes (Figs 7A and B). The typical P-TACC3 staining was restored at spindle poles after expression of YFP-TPX2 in TPX2-depleted oocytes (Fig 7A), with a substantial variability, as revealed by the quantitative analysis (Fig 7B). In contrast, the injection of YFP-TPX2ΔN in TPX2-depleted oocytes led to the formation of large microtubule structures lacking P-TACC3 labelling of their poles (Figs 7A and B). To confirm the inhibition of P-TACC3 accumulation in TPX2-depleted oocytes, we also analyzed its total amount by immunoblotting. The substantial inhibition of TPX2 increase in siTPX2-5h-injected oocytes was associated with a loss of P-TACC3 accumulation (Fig 7C).

The effect of TACC3 depletion was then investigated. In order to maximize the efficiency of the depletion, in the light of TACC3 being already present in prophase, oocytes injected with the siTACC3 were maintained in prophase for a prolonged period of time (30 hours) before meiotic maturation was allowed to proceed. In these conditions, polar body extrusion was inhibited in 31% of the oocytes (n = 103), compared to 8% in the siControl-injected oocytes (n = 39). Control oocytes that were arrested in MI had normal looking spindles (data not shown). In contrast, almost all (93%) of the siTACC3 injected oocytes arrested at meiosis I contained abnormal spindles, strikingly resembling the structures observed in TPX2 depleted oocytes expressing YFP-TPX2ΔN (Fig 7A bottom lane). Furthermore, these structures were characterized by a lack of P-TACC3 labelling at the poles (Fig 7A bottom panel and 7B), indicating an efficient depletion of TACC3 in this population of oocytes. Taken together, our data indicate that in mouse oocytes, the N-terminal and Aurora A activating domain of TPX2 is of central importance for the phosphorylation and activation of TACC3.

## Discussion

### TPX2 meiotic cell cycle regulation

We here show a unique cell cycle regulation of TPX2 in meiosis. In somatic cells, TPX2 protein levels are maximum at G2/M and drop as cells exit mitosis [11]. In contrast, TPX2 is not detected at prophase I in the oocyte. It starts to accumulate at meiosis I onset and does not drop after meiosis I exit, reaching its maximum levels in meiosis II only. The anaphase-promoting complex/cyclosome (APC/C) activator cdh1 triggers TPX2 proteolysis at the end of mitosis [13]. APC^cdh1^ was also shown to be active during prophase and prometaphase I in mouse oocytes and to induce cyclin B1 degradation in order to maintain the prophase arrest and to further slow the progression of meiosis I [20], [23]. We show here that depletion of cdh1, using antisense morpholino oligonucleotides, induced premature TPX2 accumulation in prophase-arrested mouse oocytes. Taken together, our observations strongly suggest that APC^cdh1^ dependent degradation of TPX2, governs the gradual accumulation of TPX2 in maturing oocytes, similarly to cyclin B1. Therefore our work links for the first time progression of the cell cycle to progression of spindle morphogenesis via the accumulation of a cytoskeleton component, TPX2.

### TPX2 is required for meiotic acentriolar spindle formation

Our results indicate that TPX2 is required for meiotic spindle assembly in mammalian oocytes. TPX2 depletion triggers a dose-dependent reduction in microtubule assembly and results in the formation of structures varying from mini-bipolar spindles to tiny asters associated with the chromosomes. These observations are consistent with the role of TPX2 as a central microtubule assembly factor in mitotic cells [11], [12], [16]. In addition, we observed a compaction and clustering of the chromosomes in meiosis I upon TPX2 depletion. This effect was not observed in other experimental systems [11], [12], [27] but is reminiscent of the consequence of microtubule depolymerisation induced by nocodazole in the oocyte [19]. Hence, maintaining the individual morphology of bivalent chromosomes requires the continuous assembly of TPX2-dependent microtubules during meiosis I.

### TPX2 and meiosis I versus meiosis II spindle assembly kinetics

This peculiar meiotic cell cycle regulation, as well as the functional analysis we performed, implies that meiosis II requires higher TPX2 protein levels than meiosis I. TPX2 is a key RanGTP target [14], [32]. In maturing oocytes, RanGTP is generated as a gradient around the chromosomes during both meiosis I and II. However, experimental alterations of the RanGTP gradient fully inhibit meiosis II but not meiosis I spindle assembly [8]. The results we present are consistent with this unexpected observation. Moreover, based on our results, we propose that in the oocyte, in contrast to mitotic systems, the availability of Ran effectors rather than RanGTP itself, are rate limiting for the Ran-mediated spindle assembly. We are currently trying to understand if such regulation accounts for other Ran effectors that may be involved in meiotic spindle assembly in the oocyte.

Our work also suggests that meiosis I progression requires a gradual increase in TPX2 protein levels. Interestingly, this regulation is similar to CDK1-cyclin B1 kinase activity which progressively increases via the accumulation of cyclin B1 during meiosis I [33]. In mitotic systems, the transition from interphase to M phase is associated with massive activation of CDK1-cyclin B which triggers a switch from stable to highly unstable microtubules [34]. In this context, TPX2 activity as a microtubule assembly factor might be critical to counteract the global microtubule destabilization in the cytoplasm in order to allow spindle assembly. During meiosis I, the progressive activation of CDK1-cyclin B1 may not trigger such a sudden but rather induce a progressive increase in microtubule dynamics. TPX2-dependent microtubule assembly would be gradually required and therefore tightly controlled both temporally (by increased protein levels) and spatially (by the RanGTP gradient). The fact that TPX2 over-expression at meiosis I onset induces the formation of large unorganised microtubules structures supports this hypothesis. Central to this process, the APC^cdh1^ activity during prometaphase I would in parallel monitor cyclin B1 and TPX2 accumulation and coordinate microtubule assembly to the increase in microtubule dynamics.

In addition, the notion that TPX2 premature expression induces the formation of unorganised microtubule structures also suggests that some microtubule associated proteins or molecular motors need to become only progressively available in the cytoplasm to organize the microtubule network during meiosis I. Similarly to TPX2, the kinesin HSET [35] and the protein MISS, a substrate of MAPK which is required for proper meiosis II spindle morphology [36], have been shown to be up-regulated during mouse oocyte meiotic maturation. In addition, the anchoring and stabilization of microtubules at the kinetochore plate is a late event of Meiosis I [5], suggesting a late activation of the factors involved in this interface. The limiting factors that govern the kinetics of microtubule assembly and organization into a functional spindle during meiosis I remain to be identified.

### Distinct TPX2 activities are required for meiotic spindle formation

TPX2 was shown to induce a RanGTP dependent nucleation of microtubules [11]. In *Xenopus* egg extract, this nucleation is supported by a large C-terminal domain of the protein [27]. Our results indicate that this domain is indeed sufficient to achieve TPX2-dependent microtubule nucleation in maturing oocytes. In TPX2-depleted oocytes, the expression of TPX2ΔN, lacking the N-terminal 118 amino acids, is sufficient to restore both spindle size and the morphology of chromosomes (Figs 5B, 5D and 7A).

**Figure 8:**
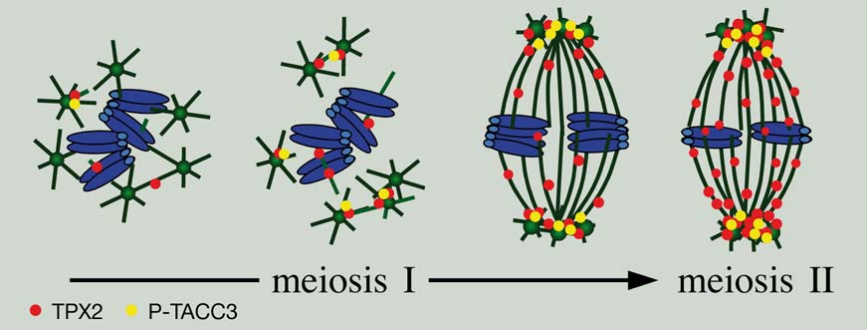
A model for TPX2 function in mouse oocytes. TPX2 (red dots) accumulates during meiotic maturation. It induces microtubule assembly around the chromosomes. In parallel, it locally activates TACC3 (yellow dots) in the vicinity of MTOCs (green discs), which in turn stimulates spindle pole stability. Chromosomes appear in blue, microtubules in green and MTOCs as green discs.

However, our results also indicate that the N-terminal domain of TPX2 is essential for an activity, which is strictly required for maturation: oocytes depleted of endogenous TPX2 but expressing TPX2ΔN remain arrested in meiosis I and exhibit dramatic spindle pole defects. It has been shown that the N-terminal domain of TPX2 binds and activates the Aurora A kinase, an essential regulator of centrosome and spindle pole assembly during mitosis. Aurora A phosphorylates and activates the centrosomal protein TACC3 resulting in the stabilization of centrosome-associated microtubules (for review see [17]). Thus, TPX2 may indirectly contribute to the integrity of centrosomal spindle poles in somatic cells. Our result clearly show that such a mechanism is at play in vertebrate oocytes lacking canonical centrosomes and containing multiple MTOCs. In maturing oocytes, while TACC3 is stably expressed, the phosphorylated form of the protein progressively accumulates concomitant with TPX2 and closely associates with MTOCs. TPX2 depletion abolishes TACC3 phosphorylation, which cannot be restored upon expression of TPX2ΔN, lacking the Aurora A-binding domain. Under these conditions, the spindle pole cohesion is weakened and multi-polar structures form. We observed strikingly similar spindle structures under conditions of TACC3 depletion. We propose that in maturing oocytes, TPX2 triggers the activation of TACC3 on MTOCs and consequently stabilizes the emanating microtubules that could “cross-link” distinct MTOCs at the pole in order to maintain acentriolar spindle pole integrity (see Fig 8). In conclusion, TPX2 is a key contributor of meiotic acentriolar spindle assembly by two distinct activities, *i.e.* (1) promoting microtubule assembly and thus controlling spindle volume and (2) ensuring MTOCs cohesion and spindle pole integrity (Fig 8).

### Note added in proof

Recent work by Sardon [37], using *Xenopus* egg extracts, supports the indirect role of TPX2 through Aurora A in the local activation of TACC3 on centrosomes.

## Supporting Information

Video S1 Time-lapse microscopy of mouse oocytes injected with a cRNA encoding YFP-TPX2, expressed at low levels. Up is the transmitted light and bottom corresponds to the YFP signal. Images were taken every 15 minutes. Times after GVBD are indicated in the lower left corner.
(0.25 MB MOV)

Video S2 Time-lapse microscopy of mouse oocytes injected with a cRNA encoding YFP-TPX2, expressed at high levels. Up is the transmitted light and bottom corresponds to the YFP signal. Images were taken every 15 minutes. Times after GVBD are indicated in the lower left corner.
(0.38 MB MOV)

Video S3 Time-lapse microscopy of β-Tubulin GFP expressing mouse oocytes injected with siCo-5h. Images were taken every 20 minutes. Times after GVBD are indicated in the lower left corner.
(0.03 MB MOV)

Video S4 Time-lapse microscopy of β-Tubulin GFP expressing mouse oocytes injected with siTPX2-5h. Images were taken every 20 minutes. Times after GVBD are indicated in the lower left corner.
(0.06 MB MOV)

Video S5 Time-lapse microscopy of histone-RFP expressing mouse oocytes injected with siCo-5h. Images were taken every 20 minutes. Times after GVBD are indicated in the lower left corner.
(0.20 MB MOV)

Video S6 Time-lapse microscopy of histone-RFP expressing mouse oocytes injected with siTPX2-5h. Images were taken every 20 minutes. Times after GVBD are indicated in the lower left corner.
(0.04 MB MOV)

Video S7 Time-lapse microscopy of mouse oocytes expressing YFP-TPX2 and injected with siTPX2-5h. Images were taken every 30 minutes. Times after GVBD are indicated in the lower left corner.
(0.09 MB MOV)

Video S8 Time-lapse microscopy of mouse oocytes expressing YFP-TPX2ΔN and injected with siTPX2-5h. Images were taken every 20 minutes. Times after GVBD are indicated in the lower left corner.
(0.07 MB MOV)
